# An Evaluation of the Results of the Steroid and Non-steroidal Anti-inflammatory Drug Treatments in Subacute Thyroiditis in relation to Persistent Hypothyroidism and Recurrence

**DOI:** 10.1038/s41598-019-53475-w

**Published:** 2019-11-15

**Authors:** Muhammed Erkam Sencar, Murat Calapkulu, Davut Sakiz, Sema Hepsen, Arif Kus, Pinar Akhanli, Ilknur Ozturk Unsal, Muhammed Kizilgul, Bekir Ucan, Mustafa Ozbek, Erman Cakal

**Affiliations:** 1 Department of Endocrinology and Metabolism, University of Health Sciences, Diskapi Yildirim Beyazit Training and Research Hospital, Ankara, Turkey; 2 Department of Internal Medicine, University of Health Sciences, Diskapi Yildirim Beyazit Training and Research Hospital, Ankara, Turkey

**Keywords:** Prognostic markers, Thyroid diseases

## Abstract

Subacute thyroiditis (SAT) is an inflammatory thyroid disease. The main purpose of the treatment is to relieve pain and control the inflammatory process. The aim of the present study was to evaluate the therapeutic effects of steroid and non-steroidal anti-inflammatory drugs (NSAIDs) in SAT. Initial laboratory data, treatment response, and long-term results of 295 SAT patients treated with ibuprofen or methylprednisolone were evaluated. After the exclusion of 78 patients, evaluation was made of 126 patients treated with 1800 mg ibuprofen and 91 patients treated with 48 mg methylprednisolone. In 59.5% of 126 patients treated with ibuprofen, there was no adequate clinical response at the first control visit. In 54% of patients, the treatment was changed to steroids in mean 9.5 days. Symptomatic remission was achieved within two weeks in all patients treated with methylprednisolone. The total recurrence rate was 19.8%, and recurrences were observed more frequently in patients receiving only steroid therapy than in patients treated with NSAID only (23% vs. 10.5% p:0.04). Persistent hypothyroidism developed in 22.8% of patients treated only with ibuprofen and in 6.6% of patients treated with methylprednisolone only. Treatment with only ibuprofen (p:0.039) and positive thyroid peroxidase antibody (anti-TPO) (p:0.029) were determined as the main risk factors for permanent hypothyroidism. NSAID treatment is not as effective as steroid treatment in early clinical remission. Steroid treatment was detected as a protective factor against permanent hypothyroidism. Therefore, steroid therapy may be considered especially in anti-TPO positive SAT patients and patients with high-level acute phase reactants.

## Introduction

Subacute thyroiditis (SAT) is an inflammatory thyroid disease with a background of possible viral etiology, and it manifests with clinical symptoms, including severe neck pain, fever, and fatigue^1–3^. The diagnosis of this disorder is based on physical examination, clinical symptoms, laboratory, and ultrasonographic findings. SAT mostly presents with thyrotoxicosis and is followed by hypothyroidism before complete remission^3,4^. Although SAT is a self-limiting disease within weeks, it requires treatment for the painful condition and thyrotoxicosis symptoms. The primary goal of the treatment is relief of symptoms that can be provided by beta-blocker agents, NSAIDs, and steroids. Although there is no consensus on initial therapy, the recommended approach is to start treatment with NSAIDs in mild cases and steroids in severe disease, but there are not enough data in the literature about the short or long-term consequences of these therapies^4^. The aim of this study was to evaluate and compare the short and long-term results of the initial therapies in SAT patients.

## Material and Methods

### Patients

**Figure 1:**
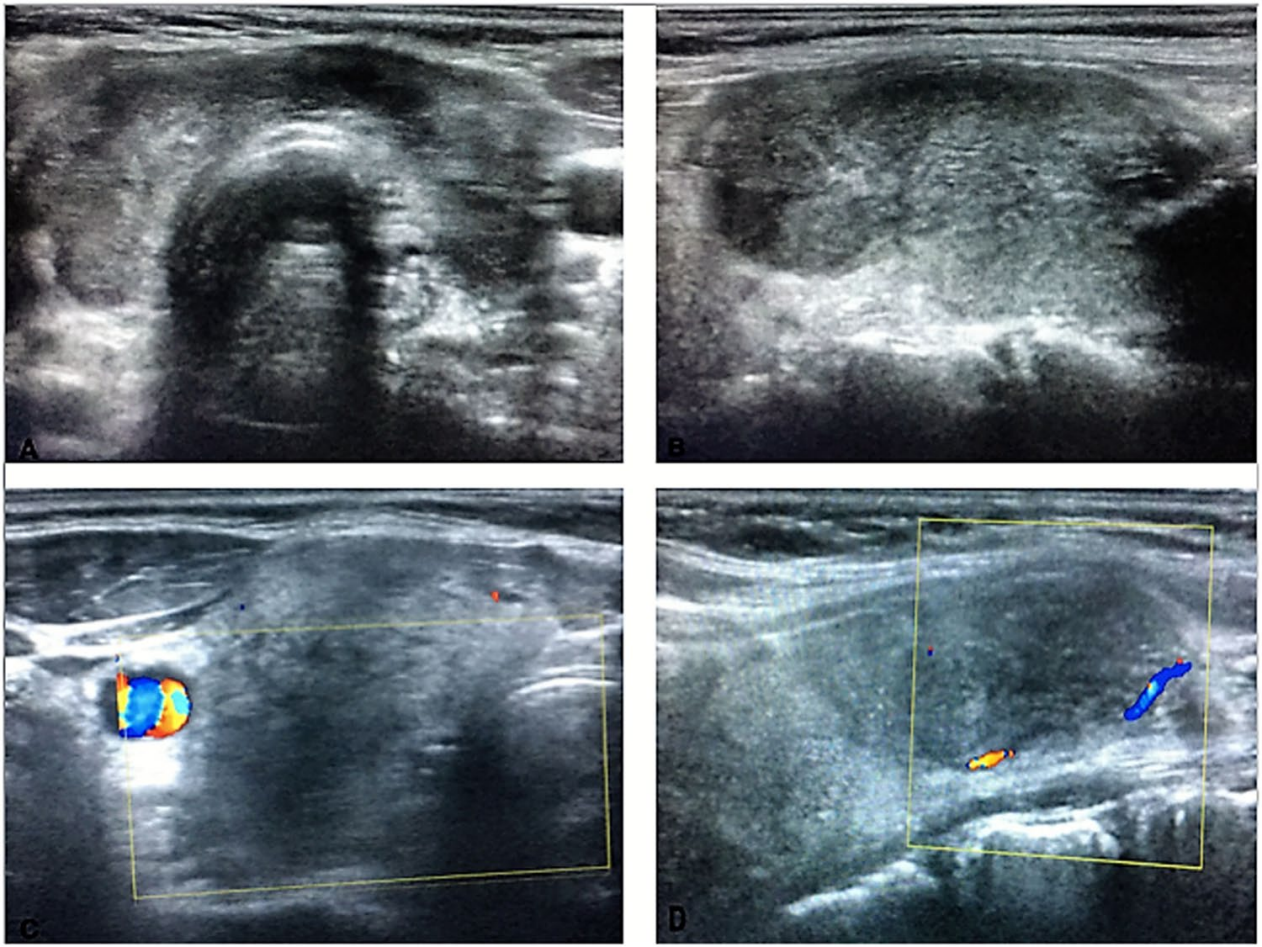
(**A**) Thyroid ultrasound typically shows a bilateral heterogeneous hypoechoic pattern, (**B**) hypoechoic heterogeneous area painful to the touch of the probe in the right lobe (**C**,**D**) shows no vascular flow in hypoechoic areas.

This retrospective study included a total of 295 patients who were diagnosed with SAT and treated at our institution between January 2014 and September 2018. This investigation was approved by the Ethics Committee of University of Health Sciences, Diskapi Yildirim Beyazit Training and Research Hospital. The study was conducted in accordance with the Declaration of Helsinki. Informed consent was obtained from each patient for participation in the study. SAT diagnosis was made by physical examination, clinical symptoms, laboratory tests; C-reactive protein (CRP) and erythrocyte sedimentation rate (ESR), thyroid function tests, and thyroid ultrasonography findings and cytopathological examination in suspected patients (Fig. 1). Patients who were followed up for less than six months, treated with drugs other than ibuprofen or methylprednisolone and patients without acute phase reactants or ultrasonography at diagnosis were excluded from the study. Acute exacerbation of chronic thyroiditis was differentiated from SAT by cytological and ultrasonographic findings, acute phase reactants, clinical symptoms, and response to steroid treatment^5^.

### Method

Subacute thyroiditis is fundamentally a clinical diagnosis and is supported with laboratory and imaging findings. According to the American Thyroid Association, there are no definitive criteria for the diagnosis of SAT^4^. As in many studies in the literature^6–8^, our patients were diagnosed with the following criteria; painful, tender, and hard goiter; elevation of ESR or CRP; elevation of serum free thyroxine (FT_4_) and suppression of serum thyrotropin (TSH); presence of hypoechoic areas with blurred margins and decreased vascularization appearance on ultrasonography of painful thyroid regions. Cytological confirmation was performed in patients with suspected diagnosis. In addition, an improvement in ultrasonographic findings was confirmed by control ultrasonography in all patients. All patients were treated with either 1800 mg ibuprofen given in 3 doses or 48 mg methylprednisolone given in 2 doses and 40 mg propranolol in 2 doses for thyrotoxicosis symptoms if needed. Treatments were selected according to the clinical experience of the attending endocrinologists. The steroid treatment protocol used in this study started with 48 mg and continued with 32, 24, 16, 8, 4 mg weekly and was completed in 6 weeks. The NSAID treatment continued until clinical symptoms disappeared and acute phase reactants normalized. The dose titration and duration of treatment could be changed according to the patient’s clinical symptoms. Clinical remission was decided by resolution of symptoms. In our clinic practice all SAT patients are questioned about clinical symptoms and simple pain intensity scale of “none, mild, moderate, and severe” is administered within the first two weeks of treatment^9,10^. The ibuprofen treatment is replaced by steroid treatment in patients without adequate regression in clinical symptoms or in patients who describe moderate or severe pain despite ibuprofen treatment. A state of permanent hypothyroidism was accepted when the hypothyroid phase of SAT persisted for at least six months and levothyroxine treatment had to be continued during this period. Patients using levothyroxine were excluded from the evaluation of permanent hypothyroidism. Recurrence was defined by the relapse of clinical symptoms with elevated ESR/CRP and ultrasonographic findings. All patients underwent an ultrasonographic examination at the time of diagnosis, after treatment was completed and at the time of relapse. US was performed by the authors experienced in ultrasonography using Hitachi HI Vision Prerius (Hitachi, Tokyo, Japan), with a linear 13 MHz probe. The thyroid volume was calculated using the ellipsoid volume formula (ml) (Length (cm) × Width (cm) × Thickness (cm) × π × 4/3). All laboratory tests were applied at the time of diagnosis, then repeated when treatment was completed, and on the occurrence of relapse. Thyroid function tests were repeated every 3 months in patients with persistent hypothyroidism and levothyroxine doses were adjusted according to TSH levels. Thyroid function tests and thyroid antibodies were measured with an automated direct chemiluminescent immunoassay (Beckman Coulter, CA, USA). Reference ranges were defined as TSH: 0.38–5.33 mIU/L, fT4: 0.60–1.25 ng/dl, fT3: 2.28–4 ng/L, anti-thyroglobulin antibody (anti-TG): 0–40 IU/mL, anti-thyroid peroxidase antibody (anti-TPO): 0–35 IU/mL, ESR: 0–20 mL/hour, CRP: 0–5 mg/L, Leukocytes: 3570–11010 10^3^/uL, Neutrophil: 1690–7550 10^3^/uL.

### Statistical analysis

Statistical analyses were performed using SPSS software (version 21.0, SPSS, Chicago, IL, USA). Categorical data were summarized with frequencies and percentages (%). All continuous variables with normal distribution were expressed as mean ± standard deviation (SD), and non-normally distributed variables were expressed as median (range) values. The Independent Samples t-test was used to compare continuous variables with normal distribution, and the Mann-Whitney *U* test was used for non-normally distributed variables. Relationships between categorical variables were tested using Chi-square analysis. Binary multiple logistic regression analysis was performed to evaluate the relationship between permanent hypothyroidism and categorical variables. (all variables displaying a significant p-value on the univariate analysis). Receiver operating characteristic (ROC) analysis was performed to determine the optimal cut-off levels in variables affecting the non-responsive treatment group. The optimal cutoff levels are the values yielding maximum sums of sensitivity and specificity from the ROC curve. A value of *p* < 0.05 was accepted as statistically significant.

### Ethical approval

The study was approved by the Ethics Committee of our institute.

### Informed consent

All participants were informed about the research protocol and all declared voluntary participation with a signed written consent form.

## Results

**Figure 2:**
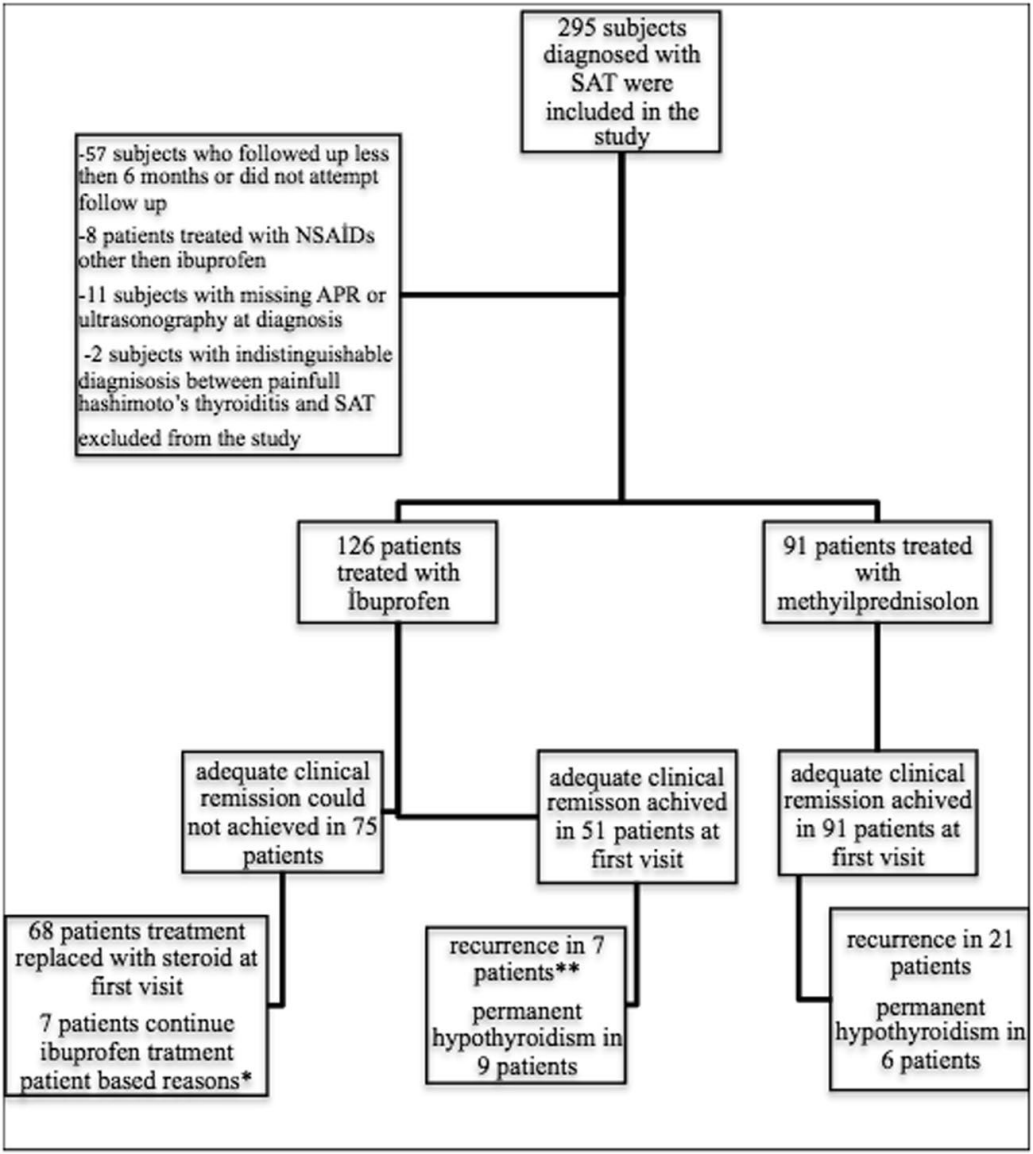
Patient selection protocol and clinical responses of patients. SAT: subacute thyroiditis, NSAIDs: non-steroidal anti-inflammatory drugs, APR: acute phase reactants, *2 patients had poor glycemic control, 5 patients did not accept steroid treatment, **6 patients treated with NSAID and 1 patient treated with steroid on recurrence time.

**Figure 3:**
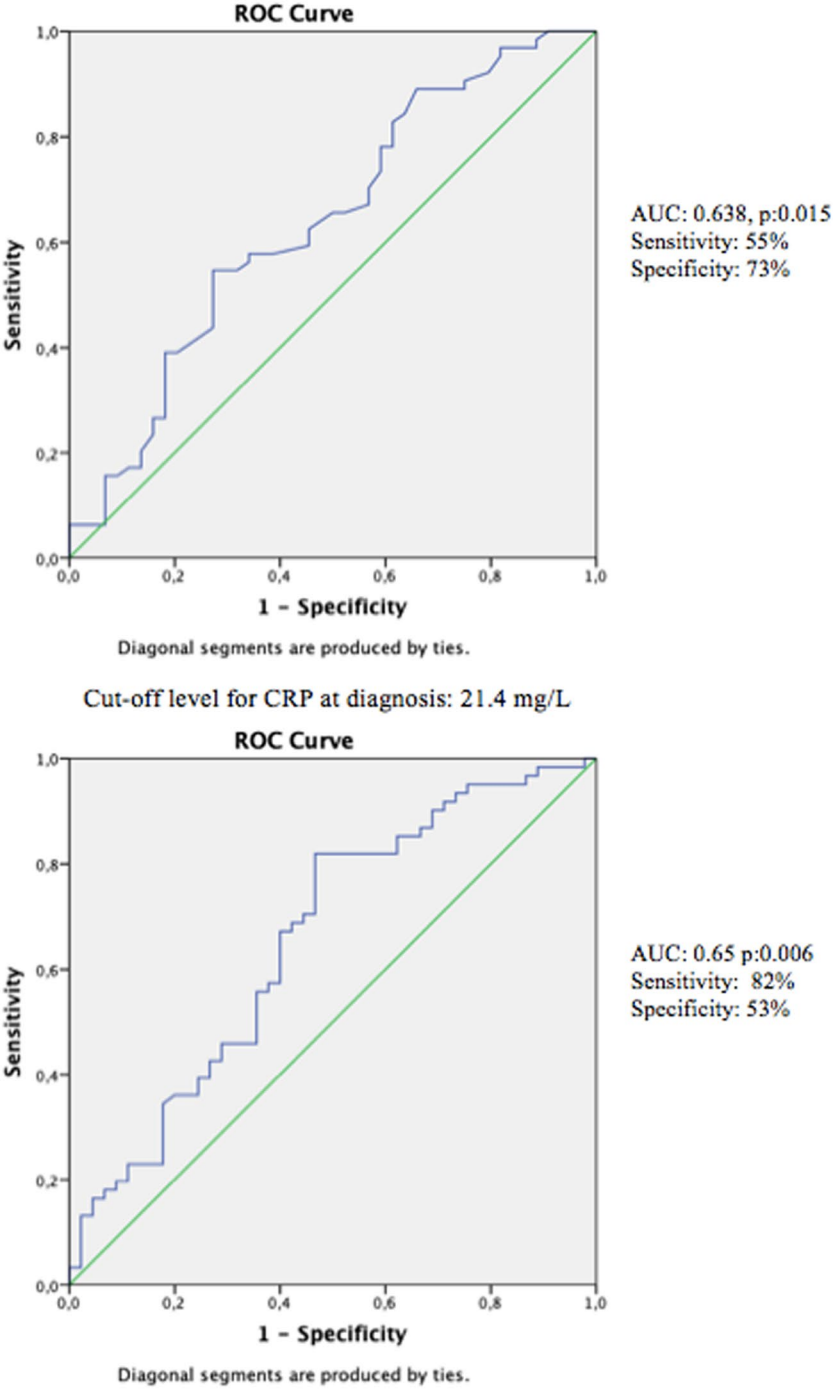
ROC curve analysis of ESR and CRP levels to identify a cut-off level for unresponsiveness to NSAID treatment. ESR: erythrocyte sedimentation rate; CRP: C-reactive protein NSAID: non-steroidal anti-inflammatory drug.

A total of 295 subjects diagnosed with SAT were enrolled in the study. Exclusions were made of 57 subjects who were followed up for less than 6 months or did not attend follow-up examinations, 11 subjects with missing acute phase reactants or ultrasonography at diagnosis, 8 subjects who were treated with drugs other than ibuprofen and 2 subjects with painful Hashimoto’s thyroiditis which could not be differentiated from SAT (Fig. 2). The median follow-up time of patients was 27 (6.2–64) months. The full demographic and baseline clinical data of the remaining 217 subjects are reported in Table 1 and the detailed clinical and prognostic information of patients receiving steroid and NSAID treatment is provided in Table 2. Treatment was applied to 126 patients with 1800 mg ibuprofen, and 91 patients were treated with 48 mg methylprednisolone. The median treatment period for the NSAID group was shorter than that of the steroid group (14 (2–42) vs. 42 (14–70) days) since the treatment was replaced with steroid in the NSAID non-responsive group. The age, ESR, CRP, free thyroxine (fT4) free triiodothyronine (fT3) and thyroid autoantibody levels were similar in both groups (p > 0.05). The thyroid stimulating hormone (TSH) level, white blood cell (WBC) and neutrophil count were significantly different between the two groups (p:0.019, p:0.003, p:0.006 respectively). In 75 (59.5%) of 126 patients treated with ibuprofen there was no adequate clinical response and in 68 (53.9%) patients the treatment replaced by steroid in mean 9.5 (2–28) days because of inadequate clinical response. ESR and CRP values were significantly higher in the ibuprofen non-responsive group compared to the responsive group (p:0.015, p:0.006, respectively) (Table 3). A negative correlation was found between NSAID treatment response and ESR (r^2^:0,072, p:0,023) and CRP (r^2^:0,077, p:0,022) levels in the univariate logistic regression analysis. The cut-off level for ESR was found to be 50 mm/hour (area under curve (AUC):0.638, p:0.015; sensitivity:55%; specificity: 73%) and the cut-off level for CRP was found to be 21.5 mg/L (AUC:0.657, p:0.006; sensitivity:82%; specificity:53%) by ROC curve analysis to identify a cut-off level for unresponsiveness to NSAID treatment (Fig. 3). The age, WBC, neutrophil count, and thyroid function tests were similar in the ibuprofen responsive group and the non-responsive group (p > 0.05). Adequate clinical remission at the first control visit was achieved in all 91 patients treated with methylprednisolone.

**Table 1:** Demographic and Clinical Data of Subacute thyroiditis patients at diagnosis.

	Total	Initial treatment: NSAIDs	Initial treatment: Steroid	p
n	217	126	91	
Female/male n	177 / 40	106/ 20	71/ 20	
Age (year)	43 ± 9	43 ± 10	43 ± 8	0.966
Follow up time (month)	27 (6.2–64)			
Treatment period (day)		14 (2–42)	42 (14–70)	0.001
Total Leukocytes (10^3^/uL)	9100 (3500–21100)	8500 (3500–21100)	9600 (5900–19000)	0.003
Neutrophils (10^3^/uL)	6100 (2700–17100)	5720 (2800–17100)	6700 (2700–15400)	0.006
ESR (mm/hour)	48 (8–120)	45.5 (8–120)	54 (9–120)	0.242
CRP (mg/L)	45.5 (2–300)	43 (2–300)	46 (3–202)	0.377
TSH (mIU/L)	0.04 (0.001–8.12)	0.06 (0.003–8.12)	0.03 (0.001–3.44)	0.019
fT4 (ng/dL)	1.9 ± 1	1.8 ± 1	1.97 ± 1	0.336
fT3 (ng/L)	4.6 (2.6–11.8)	4.4 (2.6–11.8)	4.7 (2.8–9.2)	0.799
Anti-TPO (IU/ ml)	1.2 (0.2–1078)	1.1 (0.2–217)	1.5 (0.3–1078)	0.365
Anti-TG (IU/ ml)	0.9 (0.1–581)	0.9 (0.1–139)	0.9 (0.1–581)	0.475
Anti-TPO positivity n (%)	17 (7.8%)	9 (7.1%)	8 (8.8%)	0.45
Anti-TG positivity n (%)	14 (6.5%)	7 (5.6%)	7 (7.7%)	0.24
Recurrence n (%)	43 (19.8%)	22 (17.7%)	21 (23%)	0.306
Permanent hypothyroidism n (%)	27 (12.4%)	21 (16.7%)	6 (6.6%)	0.027

Data are presented as mean ± SD, median (range); NSAIDs: Non-steroidal anti-inflammatory drugs, ESR: Erythrocyte sedimentation rate, CRP: C-reactive protein, TSH: Thyroid stimulant hormone, fT4: free thyroxine, fT3: free triiodothyronine, Anti-TPO: Anti thyroid peroxidase antibody, Anti-TG: Anti thyroglobulin antibody.

**Table 2:** Detailed clinical and prognostic data of patients treated with NSAİDs and steroids.

	NSAİDs responders	No clinical response with NSAIDs	Recurrence in NSAIDs responders	Permenant hypothyroidism in NSAIDs responders	Steroid respnders*	Recurrence in steroid responders	Permenant hypothyroidism in steroid responders
n (female/male)	51 (43/8)	75 (62/13)	7 (7/0)	9 (8/1)	91 (71/20)	21 (18/3)	6 (4/2)
Age (year)	42 ± 9	43 ± 10	42 ± 6	41 ± 7	43 ± 8	41 ± 6	46 ± 9,5
WBC (10^3^/uL)	8397 ± 1993	9227 ± 2719	7542 ± 1252	8387 ± 2187	10031 ± 2663	11242 ± 2855	10517 ± 4122
Neut (10^3^/uL)	5607 ± 1690	6406 ± 2489	4767 ± 1338	5476 ± 1604	6824 ± 2166	7940 ± 2725	6755 ± 2149
Treatment period (day)	26 (14–42)	9.5 (2–28)	28 (14–42)	21 (14–28)	42 (14–70)	42 (30–56)	42 (14–42)
ESR (mm/h)	42,5 (8–105)	51,5 (16–120)	19 (13–99)	43,5 (9–105)	54 (9–120)	54 (12–84)	68 (18–81)
CRP (mg/L)	19 (2–191)	54 (4–300)	11 (5–101)	48 (7–191)	46 (3–202)	42 (7–169)	39 (3–162)
TSH (mIU/L)	0,06 (0,003–8,1)	0,07 (0,003–7,1)	0,7 (0,04–4,3)	0,05 (0,01–8,1)	0,03 (0,0001–3,4)	0,14 (0,003–1,8)	0,04 (0,001–0,3)
fT4 (ng/dL)	1,7 ± 0,8	1,9 ± 1	1,25 ± 0,75	1,62 ± 0,72	1,97 ± 1	1,58 ± 0,66	1,94 ± 0,83
fT3 (ng/L)	4,4 (2,6–10,8)	4,4 (2,7–11,8)	5,2 (3,5–6,9)	4,3 (3,4–10,8)	4,7 (2,8–9,2)	4,9 (2,8–6,3)	3,7 (2,8–6,6)

*There was no patient without clinical response to steroid.

**Table 3:** Clinical data of non-responsive and responsive group to NSAIDs.

	Clinical response with NSAIDs	No clinical response with NSAIDs	p
n	51	75	—
Female/male n (%)	43/8 (84%/16%)	62/13 (83%/17%)	—
Age (year)	42 ± 9	43 ± 10	0.398
Total Leukocytes (10^3^/uL)	8397 ± 1993	9227 ± 2719	0.081
Neutrophils (10^3^/uL)	5607 ± 1690	6406 ± 2489	0.064
ESR (mm/h)	42.5 (8–105)	51.5 (16–120)	0.015
CRP (mg/L)	19 (2–191)	54 (4–300)	0.006
TSH (mIU/L)	0.06 (0.003–8.1)	0.07 (0.003–7.1)	0.157
fT4 (ng/dL)	1.7 ± 0.8	1.9 ± 1	0.356
fT3 (ng/L)	4.4 (2.6–10.8)	4.4 (2.7–11.8)	0.974

Data are presented as mean ± SD, median (range); NSAIDs: nonsteroidal anti-inflammatory drugs, ESR: Erythrocyte sedimentation rate, CRP: C-reactive protein, TSH: Thyroid stimulant hormone, fT4: free thyroxine, fT3: free triiodothyronine, Anti-TPO: Anti thyroid peroxidase antibody, Anti-TG: Anti thyroglobulin antibody.

The total recurrence rate was 19.8% in the whole population of the present study.The recurrence rate was significantly higher in patients treated with steroids only compared to those treated with NSAID only (23% vs. 10.5%, p:0.04) (Table 4). However, in multivariate regression analyses, steroid treatment was not found to be an independent risk factor for recurrence (p:0.24; odds ratio 1.805, %95 confidence interval 0.7 to 5.09). Initial WBC and neutrophil count were significantly higher in the recurrence group (p:0.005 and p:0.009 respectively), and fT4 levels were significantly higher in the non-recurrence group (p:0.04). No significant differences were determined in age, TSH, ESR and CRP between the recurrence and non-recurrence groups (p > 0.05).

**Table 4:** Comparison of recurrence and permenant hypothyroidism rates of patients treated with only NSAİDs and steroid.

	Patients treated with only NSAIDs	Patients treated with only steroid	p
n	57*	91	
Recurrence n (%)	6 (10.5%)	21 (23%)	0.04
Permanent hypothyroidism n (%)	13 (22.8%)	6 (6.6)	0.004

NSAIDs: Non-steroidal anti-inflammatory drugs; *One of the patients in NSAID treated group was excluded from comparison due to receiving steroid treatment after recurrence.

The total permanent hypothyroidism rate was 12.4% in the present study. Of the 57 patients who received only ibuprofen treatment, 13 (22.8%) patients developed permanent hypothyroidism, and 6 (6.6%) of 91 patients who received only steroid treatment developed permanent hypothyroidism (Table 4). Anti-TPO antibody positivity and ibuprofen treatment were found to be significant variables in the Pearson Chi-square test to determine the risks of permanent hypothyroidism (p:0.03 and p:0.04, respectively). In the binary logistic regression analyses, TPO antibody positivity (r^2^:0.401, p:0.029) and ibuprofen treatment (r^2^:0.401, p:0.039) were found to be independent risk factors for developing permanent hypothyroidism. Age, TSH, fT4, fT3 ESR, CRP, WBC at diagnosis, anti-TG antibody positivity and recurrences were not found to be risk factors for permanent hypothyroidism (p > 0.05). The median levothyroxine dose of the patients who developed persistent hypothyroidism was 100 mcg (50–125 mcg).

## Discussion

The aim of the treatment for SAT is to ameliorate the symptoms. Beta-blockers are used to control thyrotoxic symptoms and anti-inflammatory therapy for pain control. The clinical guidelines of the American Thyroid Association recommend NSAIDs for mild symptoms and 40 mg/day of prednisolone for severe disease or for patients who fail to respond to NSAID treatment^4^. However, no objective criteria for severe disease have been specified in the literature, so the decision is based on clinical experience. Recent studies have shown that steroids provide pain relief in a shorter time compared to NSAIDs^11,12^, and steroid treatment has been shown to reduce total disease duration^13^. However, there is no evidence in those studies that steroid therapy decreases recurrence or permanent hypothyroidism compared to NSAIDs in^11–13^. Furthermore even short-term oral steroid use is known to have serious risks^14^.

In the current study, pain relief within two weeks was not achieved by 60% of patients treated with ibuprofen. It was also determined that the rate of response to ibuprofen treatment decreased in patients with high ESR and CRP levels. Symptomatic relief was achieved within two weeks in all patients treated with steroids. Sato *et al*. also reported that with NSAID treatment, the time to complete recovery of initial symptoms was three weeks (14–32 days)^12^, whereas this was achieved in only one week with steroid treatment^12^. Fatourechi *et al*. also reported from their SAT database that it takes five weeks for relief of pain with NSAIDs and four days with steroids^11^. It can be seen that pain relief, which is the main goal of the therapy, generally cannot be achieved in a short time with NSAID treatment, especially in patients with high levels of ESR and CRP. Therefore, according to results of this study, steroid therapy may be preferred if there is an approximately 2.5-fold increase in ESR and a 4-fold increase in CRP levels at the time of diagnosis.

Another target of SAT treatment is to reduce recurrence and permanent damage. Although SAT is a self-limiting disease, 10–35% of patients develop a recurrence^11,12,15,16^ and 10–15% develop permanent hypothyroidism^4,11,13^. In the present study, the rates of recurrence and permanent hypothyroidism were 19.8% and 12.4%, respectively, which were compatible with the literature. An interesting and unexpected result of the study was that, a higher recurrence rate was observed in patients who received steroid treatment (23% vs. 10.5%). Although the WBC and neutrophil count was different between the recurrence and non-recurrence groups, it is difficult to consider that there is a relationship between recurrence rate and disease severity since the initial ESR and CRP levels were not different between the groups. Mizukoshi *et al*. also reported that it is not possible to discriminate recurrence and non-recurrence patients according to their initial clinical severity^15^. A few studies have reported that recurrence is common with steroid treatment^11,15,16^. However, the cause of SAT recurrence is still unknown, and in literature, only short-term steroid therapy and HLA haplotype have been found to be associated with recurrence^8,17^. In the current study, the treatment protocol gave a relatively short time for tapering the steroid treatment to a low dose, which may not have reflected the high recurrence rate because the mean NSAID treatment period was not longer than the mean steroid treatment period. The deleterious effects of steroids on viral replication and clearance^18^ can be hypothesized as a common cause of recurrence in SAT patients. In support of this idea, viral pericarditis patients with increased recurrence rate after steroid treatment have been reported in the literature^19,20^. In these studies, frequent recurrence is attributed to the effect of steroids on viral replication.

In the current study, the rate of persistent hypothyroidism was significantly higher in patients treated with NSAID only compared to patients treated with steroids only. As a result of this study, ibuprofen treatment and anti-TPO positivity were detected as independent risk factors for permanent hypothyroidism and contrary to expectations, the recurrence rate or initial TSH, ESR and CRP levels were not detected as risk factors for permanent hypothyroidism. Increased frequency of thyroid antibody positivity has been recently shown in SAT patients^7^, and autoimmunity has been found to increase the risk of persistent hypothyroidism in some previous studies^21–23^. In a study conducted with 103 patients, the rate of permanent hypothyroidism was 11%, and 72% of these patients had thyroid antibody positivity^21^. Benbassat *et al*. also reported that 10% of SAT patients developed permanent hypothyroidism, 33% of which were anti-TPO positive^13^. Although the prednisone group had more severe clinical features in that study, there were no differences between the two treatment groups in respect of the hypothyroidism rate. In the present study, for the first time in literature, NSAID therapy was found to be a risk factor for permanent hypothyroidism. Therefore, in SAT patients with positive thyroid peroxidase antibody, steroid therapy may be preferred to prevent permanent hypothyroidism. In contrast to the current study, Fatourechi *et al*. reported that steroid treatment had no protective effect against permanent hypothyroidism, and the risk of permanent hypothyroidism in SAT patients was found to be significantly higher with steroid therapy^11^. This was attributed to greater severity of disease in that group. However, in the current study, the duration of high-dose steroid therapy was longer than in the protocol of that study, and more patients were treated with steroid than in that study.

This study had several limitations, primarily that it was a retrospective study. Patients were assigned to the steroid or NSAID treatment groups according to the attending endocrinologist’s clinical practice, although no significant difference was found between the treatment groups in respect of the initial acute phase reactants. It is probable that patients with severe symptoms were more likely to be treated with corticosteroids. In addition, the time period between the onset of symptoms and diagnosis was not known, and as our center is a referral hospital, it is possible that some patients with mild disease and patients with low acute phase reactants at the time of diagnosis had experienced the severe period of the disease before the diagnosis. The lack of data about the side-effects of NSAIDs and steroids is another limitation of the study.

## Conclusion

In conclusion, NSAIDs fail to provide clinical remission in more than half of SAT patients, and symptomatic response to NSAIDs is lower in patients with higher ESR and CRP levels. Despite the high recurrence rate was observed in steroid-treated SAT patients, steroid treatment appears to be protective against permanent hypothyroidism. Steroid therapy should therefore be considered, especially in anti-TPO positive SAT patients and patients with high-level ESR and CRP.
